# Prediction interval: A powerful statistical tool for monitoring patients and analytical systems

**DOI:** 10.11613/BM.2024.020101

**Published:** 2024-04-15

**Authors:** Abdurrahman Coskun

**Affiliations:** Department of Medical Biochemistry, Acıbadem Mehmet Ali Aydinlar University, School of Medicine, Istanbul, Turkey

**Keywords:** analytical performance, monitoring, personalized reference interval, prediction interval, reference change value

## Abstract

Monitoring is indispensable for assessing disease prognosis and evaluating the effectiveness of treatment strategies, both of which rely on serial measurements of patients’ data. It also plays a critical role in maintaining the stability of analytical systems, which is achieved through serial measurements of quality control samples. Accurate monitoring can be achieved through data collection, following a strict preanalytical and analytical protocol, and the application of a suitable statistical method. In a stable process, future observations can be predicted based on historical data collected during periods when the process was deemed reliable. This can be evaluated using the statistical prediction interval. Statistically, prediction interval gives an *“interval”* based on historical data where future measurement results can be located with a specified probability such as 95%. Prediction interval consists of two primary components: (i) the set point and (ii) the total variation around the set point which determines the upper and lower limits of the interval. Both can be calculated using the repeated measurement results obtained from the process during its steady-state. In this paper, (i) the theoretical bases of prediction intervals were outlined, and (ii) its practical application was explained through examples, aiming to facilitate the implementation of prediction intervals in laboratory medicine routine practice, as a robust tool for monitoring patients’ data and analytical systems.

## Graphical abstract

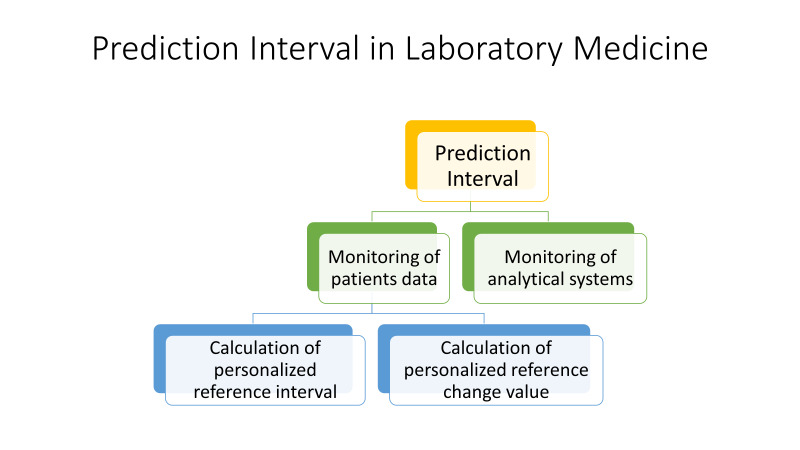

## Highlights

• Prediction interval has a great potential to be used in laboratory medicine

• It is a powerful tool for computing personalized reference interval and reference change value

• It can be used to assess the stability of analytical systems

• It can be used in monitoring the accuracy and reproducibility of analytical systems

## Introduction

Monitoring is a systematic approach dedicated to the consistent collection, analysis, and interpretation of data to generate useful information regarding the progress and performance of a specific process or system. It is based on replicate measurements and requires strict protocols and appropriate statistical techniques for collecting and analyzing data. For patients, monitoring is critical for evaluating the effectiveness of the treatment and prognosis of the diseases, and for analytical systems, it is necessary to assess the stability of measurement systems to report correct and compatible patient test results (*1*, *2*).

In medical laboratories, the performance of measurement systems is monitored using internal quality control (IQC) and external quality assessment scheme (EQAS) materials and appropriate statistical techniques (*3*, *4*). Monitoring analytical systems provides valuable information to evaluate the stability and performance of analytical systems over a long time period. The technical dysfunction of analytical systems can cause serious errors in patients’ test results. Similarly, monitoring individuals’ laboratory test results provide valuable information to evaluate the health status of individuals such as the effectiveness of treatment, side effects of drugs, prognosis of diseases, *etc.* In the monitoring of individuals’ data or analytical systems, the use of appropriate statistical techniques is as important as data collection.

Various statistical methods such as reference change value (RCV), correlation analysis or non-linear approaches have been used in the monitoring of patients’ test results or analytical systems (*5*-*7*). With prediction interval (PI), briefly, we use the past data to make a prediction for the future (*8*). Prediction interval is a powerful tool, particularly in the monitoring of processes including measurement systems and patients’ serial data.

In this paper, the theoretical, mathematical, and statistical aspects of PIs across various scenarios, specifically focusing on its application in monitoring measurement systems and patients’ serial measurement results were summarized. Practical examples were added to facilitate the implementation of PIs in laboratory medicine routine practice.

## Prediction interval

The PI is among the three recognized types of statistical intervals, which also include the tolerance interval and the confidence interval (*8*). In PI, the data collected from a measurement system is used to predict the future observations with a given probability. In other words, roughly we use the past data to estimate the future ones. If the conditions for obtaining new data are the same or similar as the obtaining past data, then the interval for new observations can be predicted with a known probability (*8*). Using past data, an interval can be calculated to predict a) a single future measurement result, b) a group of future measurement results, c) the mean of the future samples, and d) the standard deviation of future samples. However, for simplicity and practicality we focused on the estimation of an interval for a single future observation (*8*, *9*).

## Prediction interval for a single measurement result

The general equation (Eq.) to predict the interval for a single future observation is given below:


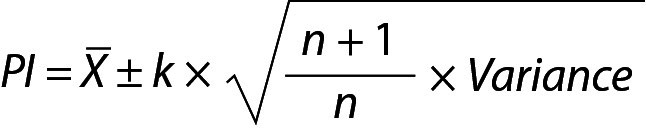
,

where X is the mean of the repeated measurements, k is a constant depending on the distribution types used to estimate the PI, variance is the square of standard deviation (SD^2^) and n is the number of previous measurements results included in calculations respectively.

The Eq. 1 is characterized by two primary components: mean and variance. Therefore, the PI for a single future measurement outcome can be estimated under four distinct scenarios (*8*, *9*).

## Scenario 1. Prediction interval estimated using the data with “population mean and population variance”

If the mean and variance of the dataset is known (population mean (µ) and variance (σ^2^)) then the PI based on normal distribution N(µ, σ^2^) can be used to estimate the future observation (x) as given below.

The z value can be calculated for lower limit (LL), x and upper limit (UL) and x will be within the interval, *i.e.* LL < x < UL and



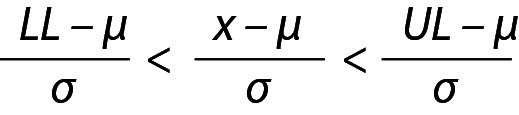





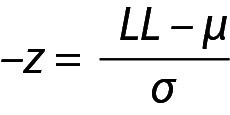




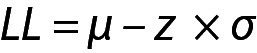
,

and similarly for UL,



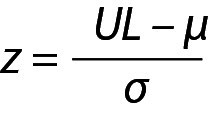




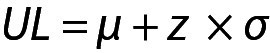
,

where z is a constant and for 95% probability it is 1.96 (≈ 2.00), µ and σ are the mean and the SD of the population respectively.

From these equations the PI for the data with population mean and variance can be written as given below:


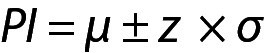
.

Note that in this model, due to the large value of n, ((n+1) / n)^1/2^ from Eq. 1 was approximated as 1.

## Practical example 1: Monitoring of analytical systems based on a large sample size

This situation arises when the instrument is in routine practice. To utilize PI for this model, a minimum of 30 measurement results (population data) are required. A practical example is given below for this model (Table 1).

**Table 1:** Daily measurement results for glucose internal quality control

**Measurements (days)**	**IQC, glucose (mmol/L)**
1	4.22
2	4.27
3	4.27
4	4.22
5	4.27
6	4.16
7	4.22
8	4.33
9	4.27
10	4.22
11	4.16
12	4.22
13	4.16
14	4.11
15	4.05
16	4.22
17	4.16
18	4.22
19	4.11
20	4.33
21	4.22
22	4.27
23	4.11
24	4.16
25	4.27
26	4.16
27	4.33
28	4.33
29	4.22
30	4.16
Mean	4.21
SD	0.07
CV(%)	1.66
These data are hypothetical and have been artificially generated for use in the practical example. IQC - internal quality control. SD - standard deviation. CV - coefficient of variation.	

Since n = 30, the PI for the next observation can be estimated using the model for PI which is based on the population mean and variance, as outlined below. From Eq. 7:



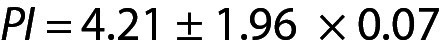




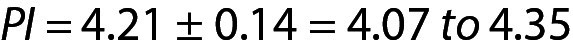
.

If the new measurement result is located within 4.07 to 4.35 such as 4.16 then it can be accepted as the expected value, otherwise if it is outside of the PI such as 4.50, it may be a random error or an indicator of unreproducible measurement.

## Scenario 2. Prediction interval estimated using the data with “sample mean and population variance”

In this case the source of variance and mean are different. The variance of the distribution is obtained from the population but the mean is estimated from the sample. Note that the term “sample” here should not be confused with a biological sample. In a statistical context, sample refers to a small group of data taken from a larger population. Statistically, a sample is a subset of the population. The greater the amount of data in the sample, the higher the precision of the mean. With the sample mean, we consider that the mean is calculated using a limited number of previous measurements and therefore it has a variation and this variation should be included in the estimation of the PI.

For a normal distribution with population variance (σ, 1) and sample mean (X) the PI (LL, UL) for future observation (x) can be calculated as given below. The mean of the sample can be estimated from the observed data:


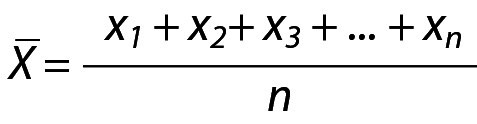
.

The variance of observation will be 1/n and the observation will have the distribution of N(µ,1/n). But on the other hand X_n+1_ has the distribution of N(µ,1.0).

The variance of the new distribution will be equal to the sum of 1 and the reciprocal of n, *i.e.*

(1 + (1/n)). The prediction distribution is N(X, 1+1/n) and the PI with 95% probability can be calculated from this distribution. The PI for sample mean and population variance can be calculated using the following equation:


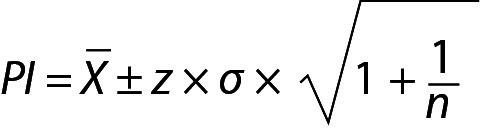
.

## Practical example 2: Personalized reference intervals based on a few measurement results

Personalized reference intervals (prRIs) can be calculated using the mean of individuals repeated measurement results (sample mean) and within-subject biological variation of the measurand (population variance). Reliable biological variation (BV) data can be obtained from the literature or European Federation of Clinical Chemistry and Laboratory Medicine (EFLM) BV database (*10*-*14*). To utilize PI for this model, a few measurement results (such as 3 or 4) are adequate. A practical example is given below for this model (Table 2). From Eq. 11:



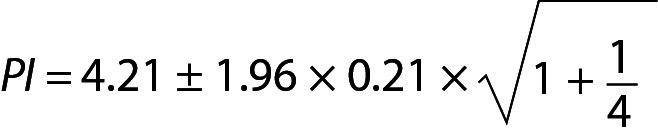




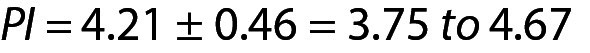
.

**Table 2:** Measurement results of glucose obtained from repeated samples taken on different days

**Measurements (days)**	**IQC, glucose (mmol/L)**
1	4.20
2	4.23
3	4.15
4	4.25
Mean	4.21
SD	0.21
CV(%)	5.00
The data in this example are hypothetical and were artificially generated for use in a practical context. IQC - internal quality control. CV - within-subject biological variation (CV_I_) of glucose and was obtained from EFLM BV database (13). SD - standard deviation derived from the within-subject biological variation (CV_I_) and the mean of the individual’s data.	

For this individual the prRI for glucose is 3.75 to 4.67 mmol/L.

## Scenario 3. Prediction interval estimated using the data with “population mean and sample variance”

In this model the variance of the distribution is estimated from the sample and therefore t distribution should be used instead of normal distribution and the PI can be calculated using the following equation:


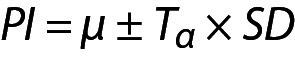
,

where *T*_α_ is the T table value (two-tailed) for n-1 freedom.

## Practical example 3

In medical laboratories the application of this model is not common. But if the mean of the measurand is known and obtained from reliable sources such as peer group (with more than 30 participants) or reference method, and the repeated measurements were performed in the laboratory to estimate the variance, than this model can be applied to predict the interval for the next measurement result (Table 3).

**Table 3:** Repeated measurement results of quality control sample for glucose

**Measurements (days)**	**QC, glucose (mmol/L)**
1	4.12
2	4.23
3	4.26
4	4.17
5	4.22
6	4.20
7	4.13
8	4.17
9	4.15
10	4.13
Mean	4.20
SD	0.05
CV(%)	1.14
The data in this example are hypothetical and were artificially generated for use in a practical context. The mean was obtained from inter-laboratory comparisons (peer group with > 30 participants). QC – quality control. SD - standard deviation. CV - coefficient of variation.	

From Eq. 14, for n = 10 the T table value for n-1 freedom is 2.26.



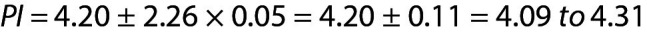



For this measurement system the PI for the next measurement result is 4.09 to 4.31 mmol/L.

## Scenario 4. Prediction interval estimated using the data with “sample mean and sample variance”

The PI for sample mean and sample variance can be calculated using the following equation:


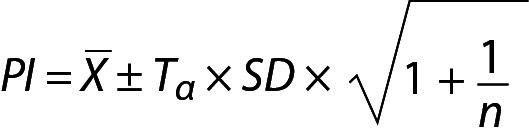
.

## Practical example 4: Monitoring of analytical systems based on a small sample size

This situation arises when a new instrument is introduced into the laboratory and requires verification before it can be used to analyze patient samples. To utilize PI for this model, a minimum of five measurement results are required. A practical example is given below for this model (Table 4).

**Table 4:** Daily measurement results of internal quality control samples for glucose

**Measurements (days)**	**IQC, glucose (mmol/L)**
1	4.16
2	4.33
3	4.22
4	4.27
5	4.22
6	4.16
7	4.33
8	4.22
9	4.16
10	4.33
11	4.16
12	4.22
13	4.33
14	4.22
Mean	4.24
SD	0.07
CV(%)	1.65
The data in this example are hypothetical and were artificially generated for use in a practical context. IQC – internal quality control. SD - standard deviation. CV - coefficient of variation.	

PI for the next observation can be estimated using the model of PI based on *sample mean and sample variance* as given below. From Eq. 16:



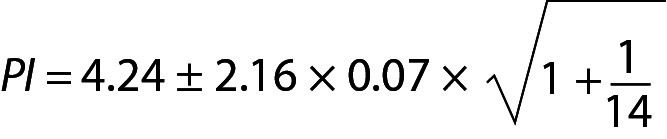




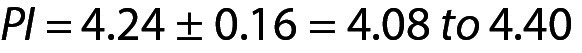
.

If the future measurement result falls outside of the PI such as 4.50, it may be a random error or an unreproducible measurement result, necessitating further verification.

## Practical example 5: Estimating personalized reference intervals using individual’s own data

For a measurand, if an individual has 5 or more repeated measurement results, both mean and variance of the measurand can be calculated using individual’s own data (*15*-*18*). The calculation of prRIs using individual’s own data for glucose and cholesterol for patient A as examples are given below (Table 5).

**Table 5:** Measurement results of glucose and cholesterol, derived from weekly samples taken from Patient A

**Measurements (Weeks)**	**Glucose** **(mmol/L)**	**Cholesterol (mmol/L)**
1	4.16	3.68
2	4.33	3.89
3	4.22	3.81
4	4.61	3.76
5	4.11	3.91
6	4.27	3.63
7	4.50	3.83
8	4.22	3.73
9	4.16	3.81
10	4.33	3.68
Mean	4.29	3.76
SD	0.16	0.09
CV(%)	3.73	2.39
The data are hypothetical and have been artificially generated for use in a practical context. SD - standard deviation. CV - coefficient of variation.		

### Step 1. Data quality assessment

Before calculation of the prRI the quality of data should be checked and confirmed (*17*). For this purpose, the data should be checked for possible outliers and then for the significance of the trend. Several statistical tests have been used for identifying outliers, and the Dixon's Q test is user-friendly for detecting potential outliers. There are no outliers in both glucose and cholesterol data according to Dixon's Q test. Regression analysis can be used to evaluate the significance of the trend. The trend is not significant in both glucose and cholesterol.

### Step 2. Calculation of the mean and variance

The square root of variance corresponds to the within-person biological variation (SD_P_). Given that the reference interval is presented in terms of absolute value, it’s preferable to calculate variations in terms of SD. While CV (%) can be determined as an alternative to SD, it would require conversion to SD. Hence, it’s more efficient to directly compute the SD.

### Step 3. Calculation of total variation around the homeostatic set point

In the example of glucose and cholesterol both mean and SD are calculated from the data of individuals (not the data of population). Therefore, the model of PI for sample mean and sample variance should be used (*16*). Using Eq. 16, for glucose:



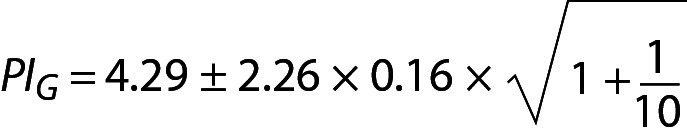




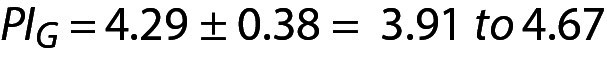
,

where PI_G_ is the PI for glucose and can be interpreted as the individual’s prRI for glucose. Similar calculations can be performed for cholesterol.



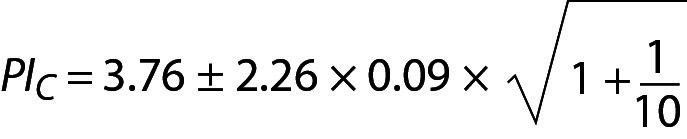




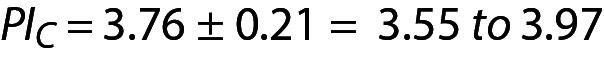
,

where PI_C_ is the PI for cholesterol. Since n = 10 for both glucose and cholesterol, T table value for n-1 freedom is 2.26. PI_C_ can be interpreted as the individual’s prRI for cholesterol.

## Confidence interval or prediction interval

**Figure 1:**
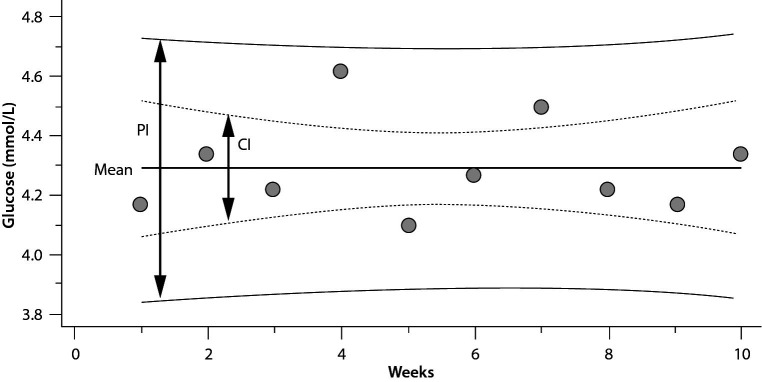
Confidence (CI) and prediction intervals (PI) for the same dataset. CI is narrower than PI.

In routine practice, it is important not to confuse the PI with confidence interval (CI) (Figure 1). Although both methods give an interval, statistically PI is different from the CI. Confidence interval gives the uncertainty of the mean *i.e.* an interval where the mean of the population can be found and can be calculated using the equation given below:


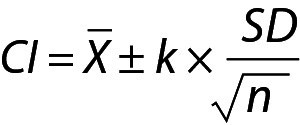
.

Note that the 4 scenarios mentioned above for PI are also applicable to CI.

## Practical example 6: Confidence and prediction intervals for glucose data given in Table 5

From Eq. 23,


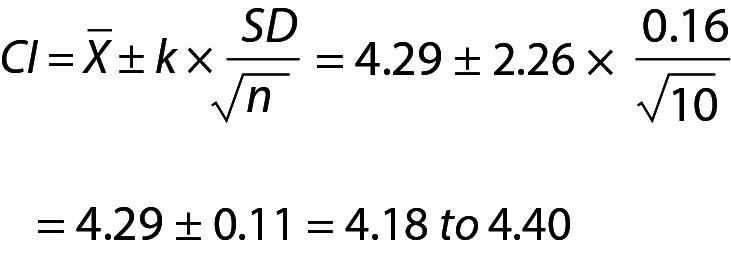
.

Since n=10, T table value for n-1 freedom is 2.26. Note that, for the same data the PI (see practical example 5 for the PI of glucose for the same data) is wider than the CI (Figure 1).

## Prediction interval for practical use

### Monitoring of analytical systems

**Figure 2:**
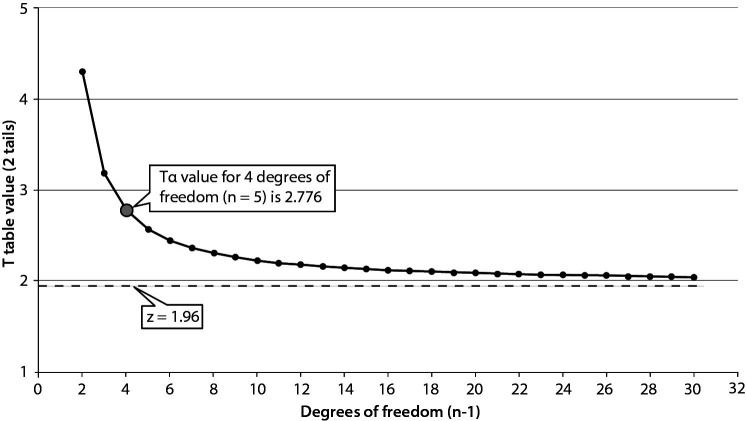
While the Tα value corresponds to the T-distribution value for (n-1) degrees of freedom, the z value is associated with the normal distribution. As the sample size increases, the uncertainty around the interval decreases.

Before introduction into routine practice, measurement instruments are monitored to evaluate the reproducibility of results and also to obtain the target value (mean) and variance of the internal quality control (IQC) samples. This period is at least 20 measurement days and known as the data collection period. It is expected to collect at least 20 data points over a 20 to 30-day period (*19*). After this period, the data is evaluated and if the instrument is stable then it is used for routine practice and patients test results are reported. In routine practice the instrument is monitored using IQC samples and the results are evaluated using Levey-Jennings chart and if the result of IQC samples are located within mean ± 2SD then it is considered as acceptable. In a statistical context, a model based on the normal distribution typically requires at least 30 data points. For a 95% probability and a bidirectional distribution, in the T-distribution, if n = 30, the T-table value for n−1 degrees of freedom will be 2.04, while the z-value for the same probability in the normal distribution is 1.96. The difference, only 4.1%, is often neglected in practice as part of a pragmatic approach. Conversely, if n = 20, the T-table value for n−1 degrees of freedom will be 2.09, while the z-value for the same probability in the normal distribution is still 1.96. This difference, amounting to just 6.6%, can also be neglected in practice, aligning with a pragmatic approach. However, if this difference is considered significant and is not overlooked, then it would be appropriate to use the T-distribution statistics rather than the normal distribution until the dataset size reaches a sufficient level to justify the use of normal distribution statistics (Figure 2).

While the term *“*prediction interval*”* isn’t directly used, the process of monitoring analytical systems essentially incorporates the concept of a PI, leveraging both population mean and population variance. If the result of IQC material is located within kSD (k is a constant and for 95% probability, it is approximately 2) then it is accepted. This situation is identical with the Eq. 7. If we have sufficient data (n ≥ 30) to calculate the mean and SD of the IQC material, then the model based on population mean and population variance can be used to predict the interval for the next measurement result.

Additionally, PI can be used to evaluate the data of new instruments that has not yet been taken into the routine practice. In installation phase, there are not sufficient data accumulated and therefore in this phase the PI based on sample mean and sample variance (Eq. 16) can be used to evaluate the data.

The main difference is that if there is sufficient data then the model based on normal distribution can be used to estimate the interval for the next measurement result and if the data is not sufficient then the model based on T distribution can be used.

### Monitoring of patients test results

The monitoring of patients test results are usually evaluated using the RCV. However, RCV usually evaluates the significance between two consecutive measurements results (*5*). The reliability of RCV have been criticized in numerous publications (*20*-*22*). As given in the following equation, the conventional RCV is calculated using the within-subject biological (CV_I_) and analytical variations (CV_A_).


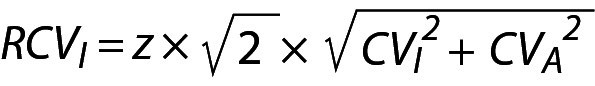
.

Note that the CV_I_ is not specific to individuals; it is calculated using data from a group of individuals. Therefore, RCV does not represent the changes in individuals’ serial measurement results. To make the RCV specific to individuals, it is recommended to use within-person BV (CV_P_) instead of CV_I_ as given below (*16*):


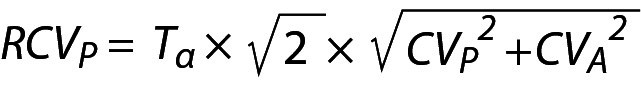
.

Since the measurement results obtained from different samples contain both analytical and biological variations then the CV of repeated measurement results can be accepted as the total CV (CV_T_) and in this scenario Eq. 26 can be simplified as follows:


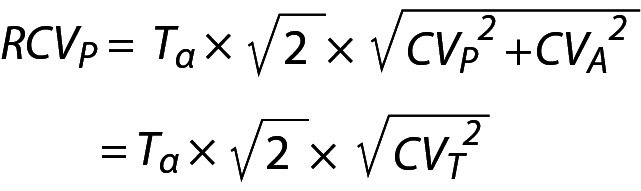
.

It should be noted that evaluation of the difference between only two consecutive measurement results may not be adequate in monitoring of patients test results. Monitoring of patients requires a comprehensive and person-specific approach. Therefore, in addition to RCV_P_, when appropriate, PI based on individuals’ repeated measurement results, *i.e.*, personalized reference interval (prRI), can also be used instead of the conventional RCV equations (*15*-*18*, *23*). For this purpose, PI derived from the data of sample mean and sample variance (Eq. 16) should be used to monitor individual’s laboratory data.

## Practical example 7: Monitoring based on reference change value derived from individual’s own data

In patient A, two measurement results for glucose and cholesterol were obtained from samples taken on days 1 and 2 (Table 6). The significance of the difference between these measurement results can be evaluated using RCV_P_ as an illustrative example. The RCV_P_ for glucose and cholesterol can be calculated from Eq. 27. The number of repeated measurement results (n) is 10, and therefore the T-table value for n-1 degrees of freedom is 2.26. For CV_P_ see Table 5.


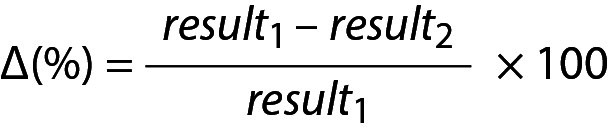
.

**Table 6:** Measurement results of glucose and cholesterol from daily samples taken from patient A

**Measurands**	**Day 1**	**Day 2**	**Population based reference interval**
Glucose (mmol/L)	4.22	5.11	3.88 - 5.55
Cholesterol (mmol/L)	3.76	4.40	2.33 - 5.18
The data in this table are hypothetical and have been artificially generated for use in a practical context.			

For glucose


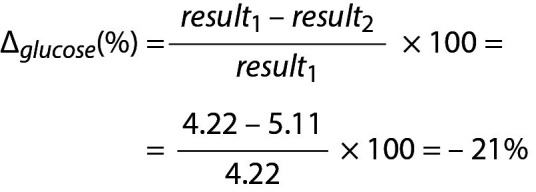
.

Due to the negative sign of the Δ*_glucose_*, the absolute value can be taken for comparison.


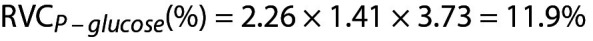
.

Since RVC*_P_*_–_ *_glucose_* < Δ*_glucose_* then the difference is significant. Note that both data (4.22 and 5.11) are located within population based reference interval but the change is significant.

For cholesterol


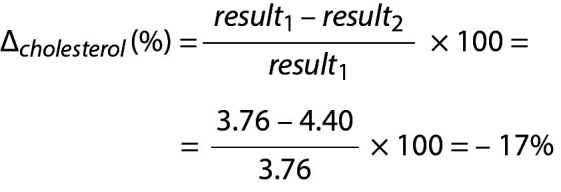
.

Due to the negative sign of the Δ*_cholesterol_* the absolute value can be taken for comparison.


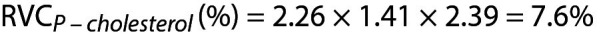
.

Since Since RVC*_P – cholesterol_*(%) < Δ*_cholesterol_* then then the difference is significant. Note that both data (3.76 and 4.40) are located within population based reference interval but the change is significant.

## Limitations

The main limitation of PI when applied in practical settings within laboratory medicine is the number of repeated measurements included in the calculations. As shown in Figure 2, the T-table value for n = 5 is 2.776, and it diminishes with an increase in n, however for the same probability (95%) the z value for normal distribution is 1.96. Increasing the number of repeated measurements reduces the uncertainty of the PI. Therefore, if feasible, it is advisable to increase the number of repeated measurements considered in the PI calculations.

## Conclusion

PI is commonly used in regression analysis but has not been used in routine medical laboratory practice. It has a great potential to be used in laboratory medicine particularly in personalized laboratory medicine. Prediction interval is a flexible statistical model and can be utilized in diverse contexts within medical laboratories. This includes computing of prRIs using a person’s prior test results and monitoring patients’ serial measurements to assess disease prognosis and treatment effectiveness. Additionally, it includes overseeing analytical systems to ensure accurate reporting of patients’ laboratory test results.

## Data Availability

No data was generated during this study.
